# Colour detection method of Korla fragrant pear based on dielectric spectroscopy technology

**DOI:** 10.3389/fpls.2025.1691673

**Published:** 2025-10-31

**Authors:** Hong Zhang, Jiean Liao, Yawen Xiao

**Affiliations:** ^1^ College of Water Resources and Architectural Engineering, Tarim University, Alaer, China; ^2^ School of Mechatronics and Automation Engineering, Xinjiang University of Technology, Kunyu, China; ^3^ School of Mechanical Engineering, Yangzhou University, Yangzhou, China

**Keywords:** dielectric spectroscopy technology, Korla fragrant pear, colour, machine learning, nondestructive testing

## Abstract

**Introduction:**

Accurate control of fruit quality determines the commercial value of Korla fragrant pear. The rapid and accurate detection of the colour of fragrant pear is crucial for improving its commercial value.

**Methods:**

In this study, a vector network analyser and coaxial probe were applied to detect the dielectric constant ϵ’ and dielectric loss factor ϵ″ of fragrant pear samples in the frequency range of 0.1–26.5GHz, and to analyse the linear relationship between the colour of fragrant pear and the dielectric parameter. Uninformative variables elimination (UVE) and the successive projections algorithm (SPA) were used to extract feature variables from the dielectric spectroscopy data; partial least squares regression (PLSR), support vector regression (SVR), and least squares support vector regression (LSSVR) were used to establish the colour prediction models of Korla f.agrant pear, respectively. The prediction results of color prediction model with full frequency band of dielectric spectrum and feature variable extraction were compared, facilitating the identification of the best prediction model.

**Results:**

The results showed that the linear correlation between ϵ’, ϵ’’ and L^*^, a^*^, b^*^ at a single frequency was weak. Both feature variable extraction methods, UVE and SPA, were able to improve the prediction accuracy of the colour of fragrant pear. The SPA-PLSR model showed the best prediction for L^*^ (R^2^ = 0.83, RMSE = 0.866, RPD = 2.477), while the UVE-PLSR model showed the best prediction for both a^*^ (R^2^ = 0.85, RMSE = 0.901, RPD = 2.523) and b^*^ (R^2^ = 0.73, RMSE = 0.895, RPD = 1.973).

**Discussion:**

The results can provide a new method for the accurate detection of the quality of Korla fragrant pear.

## 1 Introduction

Korla fragrant pears are a national geographical indication product of China, and hold the titles of “Queen of World Pears”, “Pear of Rare Quality”, and “King of Fruits” because of their aesthetic colour, sweet and smooth taste, thin skin, fine pulp, and crispy texture, with an annual production exceeding 1.5 million tons (Liu et al., 2021; Yu et al., 2023; Liu et al., 2025). In developing Asian countries, the annual postharvest loss of fruits and vegetables exceeds 50%, which has aroused widespread concern regarding food security and sustainable development (He et al., 2025). As an important index for measuring the quality of fragrant pear, colour is an important reference point for identifying fruit maturity and fruit grade and selling price (Zhang et al., 2023), which determines its commercial value (Yu et al., 2022). The evaluation of fruit colour is crucial in the sales of agricultural products (Zhang et al., 2023; Yu et al., 2022; Sanaeifar et al., 2016). Traditionally, fruit growers judge the fruit maturity and commercial value by observing the colour of fragrant pear, so it is inevitable that there will be differences in subjective judgments, which affect the reasonable quality evaluation of fragrant pear. Although machine vision and colourimeters technology can judge the colour of the fruit, these measurement results are easily affected by the lighting environment, and cannot detect other quality indexes of the fruit. Therefore, researching an accurate and efficient online detection method for the colour of fragrant pear can provide theoretical guidance for the quality control and grading of fragrant pear, and is of great significance to promote the industrial development of fragrant pear.

At present, the methods for detecting quality indexes, such as the colour of fruits, include machine vision (Liu et al., 2019; Al-Dairi et al., 2024), hyperspectral imaging (Shao et al., 2024), near-infrared spectroscopy (Alhamdan and Atia, 2017), and dielectric spectroscopy (Silva Júnior et al., 2020). For these methods, the light and background can affect the accuracy of the colour. Further, for machine vision systems, the maintenance of high-precision equipment and algorithms makes them expensive, and their versatility and adaptability are limited. Hyperspectral imaging systems have high calibration requirements, low spatial resolution, poor environmental adaptability, and high data and algorithmic complexity, limiting their applicability. Near-infrared spectroscopy is too model-dependent and sensitive to temperature and light. When the environment changes, it may lead to the spectral signal drift or distortion, which affects the detection accuracy, and the long data acquisition and processing time makes real-time detection difficult. As an emerging technology, dielectric spectroscopy technology has been applied to the detection of fruit quality indexes due to its advantages of fast, non-destructive, sensitive, efficient, and simple operation (Silva Júnior et al., 2020). used dielectric spectroscopy technology to explore the relationship between dielectric properties and the maturity of Tommy Atkins Mango, and found that the dielectric parameters were correlated with physical chemical indicators such as color of Tommy Atkins Mango during the mature stage (Krapac et al., 2024). explored the potential of electrical impedance spectroscopy as a rapid and objective technique for detecting the harvesting time of olives, and indicated that differently colored olive fruits can be classified by electrical impedancet (Tang et al., 2024). predicted soluble solid content (SSC) and hardness of fragrant pear based on dielectric spectroscopy technology and machine learning algorithms, and found that the PLSR model had the highest accuracy (Cao et al., 2023). established the relationship between dielectric properties and the internal quality of peaches based on dielectric spectroscopy and the least squares support vector machine (LSSVM) algorithm, finding that the LSSVM model predicted the quality of peaches with high accuracy (Rashvand and Soltani, 2020). used dielectric spectroscopy, artificial neural network and SVR to predict the water content of olives and found that the artificial neural network model had the best predictions. Many scholars have shown that the combination of dielectric spectroscopy with machine learning algorithms achieves good accuracy when predicting fruit quality indexes. However, most research has applied full-band dielectric spectroscopy data, which has the disadvantages of high data dimensionality, redundancy, and data noise, resulting in longer model training time, reduced model generalization ability, and difficulty in model training.

Scholars usually use feature variable extraction methods, such as SPA and UVE, for the full-band data to effectively reduce the impact of redundant dielectric spectroscopy data on the model, accelerate the computation rate, and improve the detection accuracy of the machine learning model. For example (Shang et al., 2013), established a prediction model of sugar content for nectarines based on dielectric spectroscopy with UVE and SPA feature variable extraction methods, and confirmed that the high-precision detection of the sugar content of nectarine could be realized by UVE and SPA feature variable extraction methods (Liu and Guo, 2017). established an SSC nondestructive testing model for persimmons of multiple origins using the LSSVM algorithm based on dielectric spectroscopy and the feature variable extraction method, confirming that the feature variable extraction method could improve the prediction accuracy of the SSC model for persimmons (Guo et al., 2015a). used SPA and UVE to extract the dielectric spectroscopy data of apples and combined them with extreme learning machine (ELM) and other models to predict the soluble solid content of apples. The results showed that the SPA-ELM model had the best prediction, and SPA could effectively improve the prediction accuracy of the soluble solid content of apples. These studies suggest that the prediction performance of the fruit quality index prediction model can be improved after using feature variable extraction. However, research on establishing a machine learning model based on dielectric spectroscopy and feature variable extraction to predict the colour index of Korla fragrant pear has rarely been reported, making it a significant research gap.

This study proposed an efficient method for the non-destructive detection of the color quality of Korla fragrant pears, aiming to combine dielectric spectroscopy technology and machine learning algorithms to achieve rapid and accurate prediction of pear color. The developed method not only provides a reliable technical means for pear quality evaluation but also establishes a transferable analytical framework for the non-destructive detection of other fruits and vegetables. The specific work is as follows: (1) Using a vector network analyzer and coaxial probe technology, the dielectric constant ϵ’ and dielectric loss factor ϵ’’ of pear samples were measured at 100 frequency points within the frequency range of 0.1–26.5 GHz, and the correlation between the dielectric parameters and color indicators of the pears was analyzed. (2) PLSR, SVR, and LSSVR modeling methods were used to establish prediction models for pear color. (3) The predictive performance of the three models and the accuracy of the models combined with UVE and SPA algorithms were compared and analyzed to determine the optimal prediction model for achieving accurate prediction of pear color.

## 2 Materials and methods

### 2.1 Test materials

The Korla fragrant pear samples used in this experiment were collected from a conventionally managed orchard in Block 10, Regiment 10, Alar City, Xinjiang Production and Construction Corps First Division, on October 1 and October 8, 2023. The pear trees featured uniform canopy sizes and were all 9 years old. To prevent damage and browning of the samples during collection, which could affect the experimental results, all pears were manually harvested with gloves and wrapped in foam nets. Samples were selected based on uniform size (115 ± 5 g), smooth surface, and absence of damage or disease. Fifty pears were collected on each date, resulting in a total of 100 samples for subsequent experiments. On the day of collection, the pears were transported to the Textile Engineering Laboratory of Tarim University.

### 2.2 Measurement methods

#### 2.2.1 Determination of dielectric parameters

**Figure 1:**
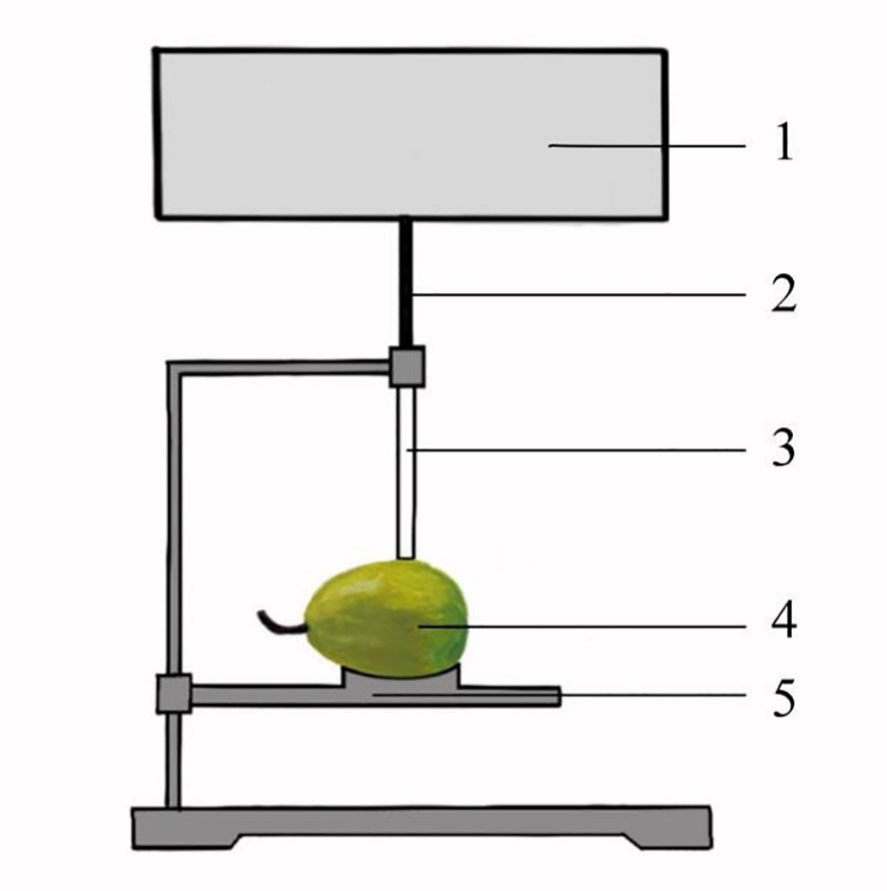
Test platform for dielectric parameters of fragrant pear. 1: Vector network analyser 2: Connecting cable 3: Coaxial probe with end opening 4: Fragrant pear 5: Lifting platform.

The vector network analyser (3671D, Siyi Science and Technology Co., Ltd. of China Electric Equipment Group, Qingdao, Shandong Province, China) and end coaxial probe were used to measure the dielectric parameters ϵ’ and ϵ’’, as shown in **Figure 1**. ϵ’ represents the ability of a dielectric material to store energy in an electric field, and ϵ′′ represents a measure of the loss energy of a dielectric material under the action of an external electric field (Nelson, 2006; Sosa et al., 2010). Before the test, the coaxial probe was connected to the preheated vector network analyser by a cable. Then, the instrument was calibrated according to open circuit, short circuit, and load standards. Finally, the frequency range was set from 0.1 to 26.5GHz, and a total of 100 frequency points were selected.

The test was carried out at room temperature, with a mean temperature of 15°C. The fragrant pear is placed on the lifting platform, and the height is adjusted to ensure close contact with the probe while preventing any damage to avoid compromising the detection results. At the largest diameter of the fragrant pear, a point was selected at 120° intervals and marked. A total of three points on each fragrant pear sample were selected as the measurement points for the dielectric parameter to measure ϵ’ and ϵ’’, and the arithmetic mean value of the three measurement results was taken as the dielectric parameter of the fragrant pear.

#### 2.2.2 Determination of L^*^, a^*^, and b^*^

The CIELab system is a uniform color space based on human visual perception, proposed by the International Commission on Illumination (CIE) (Soraya, 2021; Li et al., 2023). This system quantifies color through three dimensions: L^*^, a^*^, and b^*^, and is widely used for the quantitative assessment of the appearance quality of fruits and vegetables (Zhang et al., 2023). Therefore, this study adopted L^*^, a^*^, and b^*^ as the color quality indicators of the fragrant pears, which were measured using a colorimeter (SC-10, Shenzhen 3nh Technology Co., Ltd., Shenzhen, China). Where L^*^ indicates the brightness, with a value interval of 0–100, and a larger L^*^ value indicates a higher surface brightness of fragrant pear; a^*^ indicates the red and green difference, the value interval is -128 – +127, where +a^*^ is red, -a* is green, and a larger absolute value indicates deeper red or green; b^*^ indicates the yellow and blue difference, the value interval is -128 – +127, where +b^*^ is yellow, -b^*^ is blue, and a larger absolute value indicates a deeper yellow or blue. The measurement point of the dielectric parameter is the measurement point of colour, and the three measurement points of L^*^, a^*^, and b^*^ take the arithmetic mean value as the colour data of each fragrant pear.

### 2.3 Modelling

Three modelling methods, PLSR, SVR, and LSSVR, were used to establish the colour prediction models for Korla fragrant pear. This study sets ϵ’+ϵ’’ as the input variables of the model and L*, a*, and b* as the output variables in both the training set and the test set. Randomly, 70% of the data were used as the training set and 30% as the test set.

#### 2.3.1 PLSR model

PLSR is a regression modelling method for multiple dependent variables Y on multiple independent variables X, which combines techniques such as multiple linear regression analysis, correlation analysis, and principal component analysis. PLSR extracts and maximizes the correlation between the principal components in Y and X in the modelling process. Therefore, PLSR can analyse complex datasets comprehensively, extracting key information and constructing predictive models (Ramírez-Sánchez et al., 2025). In addition, PLSR can efficiently solve the issue of multicollinearity between the dependent and independent variables for the purpose of regression modelling. PLSR also has the advantage of obtaining desirable prediction results even with a small sample size because it emphasizes the relationship between the variables rather than the sample size.

#### 2.3.2 SVR model

SVR is a machine learning method that constructs a nonlinear regression model by a kernel function and improves the trainer’s ability to handle nonlinear problems (Abdollahpour et al., 2020; Al-Rousan and Al-Najjar, 2021). It forms an “isolation band” on both sides of the linear function with a spacing of ϵ (ϵ-insensitive loss function), and samples between ϵ do not incur a loss. Only the support vectors have an effect on its function model, and the optimized model is finally derived by minimizing the total loss and maximizing the spacing.

#### 2.3.3 LSSVR model

LSSVR is an improved version of support vector machines. It changes the inequality constraints in the traditional support vector machine to equational constraints and uses the error squared and loss function as the empirical loss in the training set to convert quadratic programming into solving a system of linear equations to increase the speed and convergence accuracy of solutions (Huang et al., 2024; Liu et al., 2024).

### 2.4 Model evaluation standards

To screen the optimal prediction model, the root mean squared error (RMSE) and coefficient of determination (R^2^) were used to evaluate the model’s ability to predict colour. The lower the value of RMSE and the higher the value of R^2^, the better the prediction effect of the model. Relative percent difference (RPD) is an important metric in model evaluation for assessing prediction accuracy. A higher RPD value indicates that the model’s predictions are more precise relative to the inherent variability of the data, and the model is more stable. When RPD > 2.5, it indicates that the model possesses excellent predictive capability, high prediction accuracy, and is highly reliable (Guo et al., 2025); When 2.0 < RPD ≤ 2.5, it indicates that the model has good predictive ability and can be used for approximate quantitative predictions and trend analysis; When RPD < 1.4, it indicates that the model has poor predictive ability and can only be used for rough qualitative discrimination of samples (Wang et al., 2022). The calculation formulas for RMSE, R^2^ and RPD are as follows, respectively in Equationa 1–3:


(1) $$\text{RMSE}=\sqrt{\frac{\sum_{\text{i}=1}^{N}{\left({\text{M}}_{\text{j}}-{\text{T}}_{\text{j}}\right)}^{2}}{N}}$$

(2) $$R^{2}=1-\frac{\sum_{\text{i}=1}^{N}{\left({\text{M}}_{\text{j}}-{\text{T}}_{\text{j}}\right)}^{2}}{\sum_{i=1}^{N}({\text{M}}_{\text{j}}-\bar{{\text{T}}_{\text{j}}}{)}^{2}}$$

(3) $$\text{RPD=}\frac{\text{SD}}{\text{RMSE}}$$

where M_j_ is the measured values of data j, and T_j_ is the prediction values of data j. $\bar{{\text{T}}_{\text{j}}}$ is the mean of the measured values. N is the total number of data, and SD is the standard deviation of the analytical samples.

### 2.5 Methods for extracting feature variables

UVE and SPA are techniques used in data analysis and machine learning for identifying and selecting effective feature variables to improve the predictive performance of models. UVE aims to automatically remove “useless” variables that do not carry more information than noise, reduce the dimensionality of the data, prevent model overfitting, and improve model interpretability. UVE, based on the principles of information theory and statistics, is highly reliable and robust, and only requires input of raw data, can be easily used, and has wide applicability. The principle of the UVE algorithm first specifies the standards for variable evaluation, and the weak correlation between the variables and the feature variables in the dataset is usually eliminated by using the Pearson correlation coefficient and chi-square tests, until the decline in the number of features in the dataset reaches a preset threshold. The feature selection process is iterated until the desired features are removed, and the specific steps of the algorithm are described in a previous study literature (Centner et al., 1996).

In addition to deleting the variables with the lowest information gain or information entropy, covariance or redundant variables could also exist in the original data set, and SPA aims to minimize the covariance by screening out the variables with the least redundant information. SPA is a forward feature variable selection algorithm, which projects wavelengths to other wavelengths after projection analysis of vectors, considering the wavelength with the largest projection vector as the wavelength to be selected, and uses the correction model to select the final feature wavelength (Zhang et al., 2022). The specific steps of the algorithm are described in a previous study (Araújo et al., 2001).

## 3 Results and analysis

### 3.1 Analysis of the linear relationship between dielectric parameters and values of L^*^, a^*^, and b^*^ of fragrant pear’s colour index

**Figure 2:**
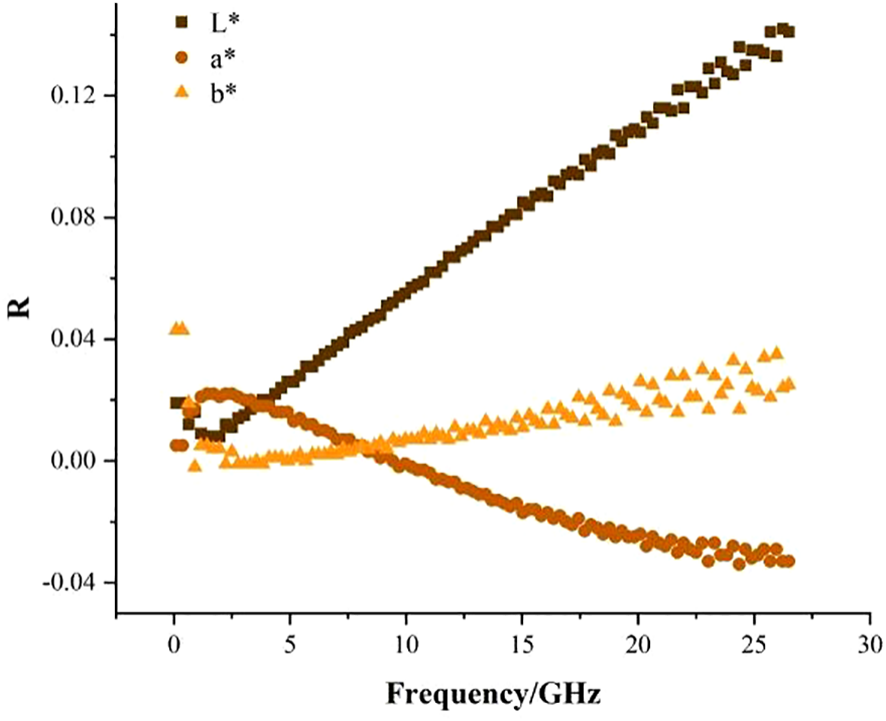
Linear relationship between ϵ' and L^*^, a^*^, b^*^.

**Figure 3:**
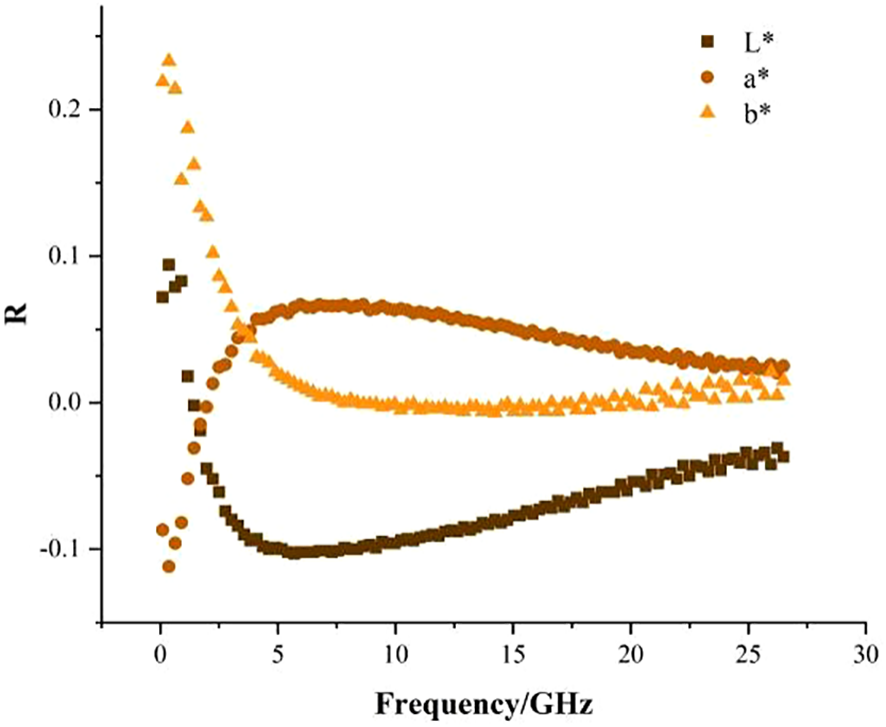
Linear relationship between ϵ'' and L^*^, a^*^, b.

The Pearson correlation analysis between the dielectric constant ϵ’ and colour indexes L^*^, a^*^, b^*^ of fragrant pear is conducted first at a single frequency. The linear relationship of ϵ’ and ϵ” with colour indexes at different frequencies is shown in **Figures 2**, **3**. As shown in **Figure 2**, L^*^, b^*^, and ϵ’ are positively correlated, and the value of the correlation coefficient is not more than 0.16. a^*^ and ϵ’ are negatively correlated, and the absolute value of the correlation coefficient is not more than 0.04, indicating that the correlation between ϵ’ and L^*^, a^*^, b^*^ is relatively small at a single frequency.

Next, this study analyses the linear relationship between loss factor ϵ’’ and colour indexes L^*^, a^*^, b^*^ of fragrant pear at a single frequency. As shown in **Figure 3**, the correlation between L^*^ and ϵ’’ changed from positive to negative, and the absolute value of the correlation coefficient was not more than 0.03. The correlation between b^*^ and ϵ’’ became weaker, and the correlation between a^*^ and ϵ’’ changed from negative to positive. Therefore, the linear correlation between ϵ’’ and L^*^, a^*^, and b^*^ is weak at a single frequency (Guo et al., 2015b). investigated the feasibility of dielectric spectroscopy as a nondestructive technique in determining SSC and firmness of pears during ripening period and found that it is impossible to select one permittivity value at a single frequency for accurate prediction of SSC and firmness of pears. This is consistent with the research conclusion of this study. The reason might be that the spatial information of the fruits obtained at a single frequency is limited (Cao et al., 2024), resulting in a poor correlation between the dielectric parameters and the color indexes.

From **Figures 2**, **3**, it can be seen that it is difficult to predict the colour of fragrant pear using dielectric parameters at a single frequency. Therefore, dielectric parameters at multiple frequencies should be used to predict the L^*^, a^*^, and b^*^ of fragrant pear.

### 3.2 Prediction of fragrant pear quality based on dielectric spectroscopy technology

#### 3.2.1 Prediction of colour indexes L^*^, a^*^, b^*^ of fragrant pear based on PLSR, SVR, and LSSVR

**Table 1** presents the results of various models for predicting the color parameters of Korla fragrant pears. The PLSR model performed best in predicting the color parameters L^*^, a^*^, and b^*^, exhibiting the highest R^2^ and RPD, along with the lowest RMSE. For the prediction of L^*^, the training set yielded R^2^, RMSE, and RPD values of 0.85, 0.753, and 2.560, respectively, while the test set produced corresponding values of 0.80, 0.962, and 2.250. For a^*^ prediction, the R^2^, RMSE, and RPD in the training set were 0.96, 0.344, and 3.965, respectively; in the test set, they were 0.80, 0.996, and 2.360. For b^*^ prediction, the PLSR model achieved R^2^, RMSE, and RPD values of 0.72, 0.890, and 1.963 in the training set, and 0.67, 0.988, and 1.734 in the test set. Furthermore, compared to the L^*^ and a^*^ parameters, all models demonstrated inferior performance in predicting b^*^. The average R^2^, RMSE, and RPD for predicting L^*^, a^*^, and b^*^ were only 0.56, 1.098, and 1.545, respectively. This may be due to the extensive redundant information contained within the dielectric spectrum data, which can impair model prediction accuracy (Guo et al., 2015a). Consequently, there remains room for improving the accuracy of PLSR models in predicting the color of Korla fragrant pears; employing feature variable extraction methods could further enhance prediction precision.

**Table 1:** Prediction results of colour indexes of fragrant pear based on PLSR, SVR, and LSSVR.

Testing index	Prediction model	Training set			Test set		
		R^2^	RMSE	RPD	R^2^	RMSE	RPD
L^*^	PLSR	0.85	0.753	2.560	0.80	0.962	2.250
	SVR	0.78	0.862	2.120	0.70	1.279	1.855
	LSSVR	0.82	0.76	2.438	0.59	1.564	1.571
a^*^	PLSR	0.96	0.344	3.965	0.80	0.996	2.360
	SVR	0.79	0.800	2.195	0.68	1.280	1.767
	LSSVR	0.83	0.723	2.514	0.64	1.280	1.723
b^*^	PLSR	0.72	0.890	1.963	0.67	0.988	1.734
	SVR	0.63	0.947	1.646	0.50	1.179	1.441
	LSSVR	0.72	0.834	1.897	0.52	1.127	1.460

#### 3.2.2 Prediction of colour indexes of fragrant pear after extraction of feature variables

##### 3.2.2.1 Prediction of colour indexes of fragrant pear based on UVE

**Table 2** shows the prediction results for the color indices of Korla fragrant pears using the UVE method. Models trained with feature variables extracted by UVE exhibited enhanced performance in predicting L^*^, a^*^, and b^*^ values. Specifically, the UVE-SVR model achieved test set R^2^ values of 0.76, 0.77, and 0.62 for L^*^, a^*^, and b^*^, respectively, with corresponding RMSEs of 0.996, 1.001, and 1.023, and RPDs of 1.999, 2.139, and 1.658. The UVE-LSSVR model yielded test set R^2^ values of 0.65, 0.71, and 0.65 for L^*^, a^*^, and b^*^, respectively, with RMSEs of 1.036, 1.044, and 0.983, and RPDs of 1.710, 1.882, and 1.726. Among the compared methods, UVE-PLSR demonstrated superior performance. For predicting L^*^, the test set R^2^, RMSE, and RPD were 0.82, 0.938, and 2.368, respectively, representing a 2.5% improvement in R^2^ over the standard PLSR model. For a^*^, the test set R^2^, RMSE, and RPD were 0.85, 0.901, and 2.523, respectively, reflecting a 6.25% increase in R^2^ compared to standard PLSR. For b^*^, the test set R^2^, RMSE, and RPD were 0.73, 0.895, and 1.973, respectively, indicating an 8.96% gain in R^2^ over standard PLSR.

**Table 2:** Prediction results of colour indexes of fragrant pear based on UVE.

Testing index	Prediction model	Selected variables	Training set			Test set		
			R^2^	RMSE	RPD	R^2^	RMSE	RPD
L^*^	PLSR	12	0.85	0.743	2.566	0.82	0.938	2.368
	SVR	13	0.75	0.947	2.068	0.76	0.996	1.999
	LSSVR	16	0.66	1.127	1.741	0.65	1.036	1.710
a^*^	PLSR	15	0.86	0.602	2.672	0.85	0.901	2.523
	SVR	29	0.88	0.586	3.045	0.77	1.001	2.139
	LSSVR	21	0.73	0.997	1.963	0.71	1.044	1.882
b^*^	PLSR	18	0.76	0.752	2.041	0.73	0.895	1.973
	SVR	5	0.66	0.920	1.759	0.62	1.023	1.658
	LSSVR	10	0.71	0.854	1.857	0.65	0.983	1.726

The results indicated that processing the dielectric spectrum data with UVE effectively improved the detection accuracy of the PLSR, SVR, and LSSVR models, with the UVE-PLSR model demonstrating the best performance in detecting the color indices L^*^, a^*^, and b^*^. Furthermore, when predicting the color index data of fragrant pears, the UVE-PLSR model extracted 12 feature variables for L^*^, 15 for a^*^, and 18 for b^*^, accounting for 6.0%, 7.5%, and 9.0% of the total input variables, respectively. This demonstrates that the UVE method can reliably identify and eliminate noise and redundant spectral variables that do not contribute to the model, thereby effectively enhancing the signal-to-noise ratio. This process simplified the model structure, reduced the risk of overfitting, and improved the model’s predictive robustness and accuracy, providing key guidance for developing low-cost detection equipment for fruits and vegetables.

##### 3.2.2.2 Prediction of colour indexes of fragrant pear based on SPA

The prediction results for the color indices of Korla fragrant pears based on the SPA method are shown in **Table 3**. When SPA was combined with PLSR to predict the color index L^*^, the R^2^, RMSE, and RPD in the test set were 0.83, 0.866, and 2.477, respectively. For predicting a^*^, the values were 0.81, 0.982, and 2.357; for predicting b^*^, they were 0.72, 0.940, and 1.924. When SPA was combined with the SVR model to predict L^*^, the R^2^, RMSE, and RPD in the test set were 0.74, 1.108, and 1.960, respectively. For predicting a^*^, the values were 0.70, 1.157, and 1.832; for b^*^, they were 0.61, 1.142, and 1.628. When SPA was combined with the LSSVR model to predict L^*^, the R^2^, RMSE, and RPD in the test set were 0.68, 1.155, and 1.774, respectively. For predicting a^*^, the values were 0.71, 0.977, and 1.913; for b^*^, they were 0.56, 1.116, and 1.536. After extracting feature variables from the dielectric spectrum data using SPA, the prediction accuracy of the PLSR, SVR, and LSSVR models was effectively improved. Among them, the SPA-PLSR model demonstrated the best performance in predicting the color parameters L^*^, a^*^, and b^*^. For predicting the L^*^, a^*^, and b^*^ values of Korla fragrant pears in the test set, the R^2^ values achieved by the SPA-PLSR model were 3.75%, 1.25%, and 7.46% higher, respectively, compared to those obtained by the PLSR model.

**Table 3:** Prediction results of colour indexes of fragrant pear based on SPA.

Testing index	Prediction model	Selected variables	Training set			Test set		
			R^2^	RMSE	RPD	R^2^	RMSE	RPD
L^*^	PLSR	20	0.85	0.767	2.629	0.83	0.866	2.477
	SVR	20	0.74	0.980	1.964	0.74	1.108	1.960
	LSSVR	25	0.68	1.139	1.780	0.68	1.155	1.774
a^*^	PLSR	20	0.89	0.586	3.030	0.81	0.982	2.357
	SVR	60	0.78	0.817	2.154	0.70	1.157	1.832
	LSSVR	60	0.83	0.803	1.781	0.71	0.977	1.913
b^*^	PLSR	15	0.80	0.691	2.267	0.72	0.940	1.924
	SVR	60	0.61	0.931	1.653	0.61	1.142	1.628
	LSSVR	60	0.63	0.950	1.658	0.56	1.116	1.536

As a forward feature selection method, SPA effectively eliminates redundant information. **Table 3** presents the number of feature variables selected by the SPA method. For predicting the color indices of Korla fragrant pears using the SPA-PLSR model, the numbers of feature variables extracted for L^*^, a^*^, and b^*^ were 20, 20, and 15, respectively, accounting for 10.00%, 10.00%, and 7.50% of the total input variables. This demonstrates that SPA processing effectively reduces data dimensionality. Furthermore, when integrated with the PLSR model, SPA selected fewer feature variables compared to SVR and LSSVR, while achieving higher prediction accuracy, further confirming the superior accuracy and robustness of the SPA-PLSR model in predicting the color indices of fragrant pears.

#### 3.3 Determination of the optimal prediction model

Both the UVE and SPA feature variable extraction methods effectively improved the prediction accuracy of the colour index of fragrant pear, and the optimal prediction results of the colour indexes of fragrant pear are shown in **Table 4**. In the prediction of L^*^, the SPA-PLSR exhibited slightly higher prediction accuracy than the UVE-PLSR, with R², RMSE, and RPD values in the test set of 0.83, 0.866, and 2.477, respectively. When predicting a^*^, UVE-PLSR had the highest R^2^ and RPD, and the lowest RMSE in the test set, at 0.85, 0.901 and 2.523, respectively. When predicting b^*^, although the accuracy of UVE-PLSR was slightly lower than that of SPA-PLSR in the training set, UVE-PLSR had a higher accuracy than the SPA-PLSR model in the test set, whose R^2^, RPD and RMSE values were 0.73 0.895 and 1.973, respectively. Overall, the prediction accuracy of the colour indexes of fragrant pear L^*^ and a^*^ are high, but the prediction accuracy of b^*^ needs to be improved.

**Table 4:** Optimal prediction results for the colour indexes of fragrant pear.

Testing index	Prediction model	Selected variables	Training set			Test set		
			R^2^	RMSE	RPD	R^2^	RMSE	RPD
L^*^	UVE-PLSR	12	0.85	0.743	2.566	0.82	0.938	2.368
	SPA-PLSR	20	0.85	0.767	2.629	0.83	0.866	2.477
a^*^	UVE-PLSR	15	0.86	0.602	2.672	0.85	0.901	2.523
	SPA-PLSR	20	0.89	0.586	3.030	0.81	0.982	2.357
b^*^	UVE-PLSR	18	0.76	0.752	2.041	0.73	0.895	1.973
	SPA-PLSR	15	0.80	0.691	2.267	0.72	0.940	1.924

## 4 Discussion

In this study, dielectric spectroscopy was combined with the feature variable extraction method to establish a machine learning prediction model for the colour of Korla fragrant pear. The optimal prediction model for L^*^ was SPA-PLSR (with R^2^ of 0.83, RMSE of 0.866, and RPD of 2.477), the optimal prediction model for a^*^ was UVE-PLSR (with R^2^ of 0.85, RMSE of 0.901, and RPD of 2.523), and the optimal prediction model for b^*^ was UVE-PLSR (with R^2^ of 0.73, RMSE of 0.895, and RPD of 1.973) (Xia et al., 2025). combined near-infrared spectroscopy with UVE and SPA to establish a PLSR prediction model for the colour of Korla fragrant pear, and found that the UVE and SPA feature extraction variable methods can effectively improve the prediction accuracy of the PLSR model, which is in line with the conclusions of this study. The R^2^ of the optimal model for predicting L^*^, a^*^, and b^*^ were 0.64, 0.71, and 0.71, and the RMSE of the optimal model were 1.19, 1.28, and 1.25, respectively. However, this study used dielectric spectroscopy technology to predict the colour of fragrant pear, which had a higher accuracy. Therefore, dielectric spectroscopy technology may be more suitable for detecting the colour indexes of fragrant pear. In addition, previous studies have shown that dielectric spectroscopy technology can also be used for the online detection of fruit quality indexes, such as the sugar degree (Hua et al., 2024), hardness (Guo et al., 2015b), and SSC (Cao et al., 2024). In comparison to machine vision and colourimeters technology, the use of dielectric spectroscopy technology in the online detection of multiple quality indexes of fruits is more in line with the production needs.

The combination of nondestructive testing technology and feature variable extraction methods (such as UVE and SPA) can effectively improve the accuracy of machine learning models to predict the fruit quality (Li et al., 2020; Gao et al., 2025; Hu et al., 2019). The feature variables with the best predictive ability can be selected after processing using the feature variable extraction method, which reduces the model complexity and information overlap, and improves the prediction accuracy and stability of the model (Meng et al., 2025). combined hyperspectral imaging with various algorithmic models, such as UVE, SPA, and PLSR, to nondestructively test the soluble solid content of multi-species blueberries, finding that the UVE-PLSR prediction model had the highest accuracy (Che et al., 2024). combined near-infrared spectroscopy with UVE and SPA to extract effective wavelengths, and the results showed that the model established by PLSR predicted the hardness and SSC of fragrant pear better (Tang et al., 2024). predicted SSC and hardness of fragrant pear based on dielectric spectroscopy technology and machine learning models, and found that compared with the SVR and PSO-LSSVR models, the PLSR model had the highest accuracy (Tian et al., 2024). used PLSR and random forest algorithms to establish quantitative prediction models for SSC and random forest based on the bulk optical properties of apples, and indicated that the PLSR models were optimal for quantitative prediction of SSC and fruit firmness. This study also shows that the colour prediction of fragrant pear established by UVE- PLSR and SPA- PLSR was better, which suggests that the PLSR is more effective for establishing fruit quality prediction models. It may be that PLSR model can effectively deal with the problem of covariance and construct a stable prediction model even if the independent variables are highly correlated with each other.

This study merely provides an new detection method for the colour of fragrant pear based on dielectric spectroscopy combined with machine learning models. This methodology can be broadly scaled up to diverse sectors in agricultural testing, offering novel approaches for enhancing food safety and quality surveillance. To achieve practical application, further improvement is still needed. Further, differences in the growth environment and planting management mode will lead to differences in samples of fragrant pear from different origins. The fragrant pear used in this study are from the First Division of Xinjiang, so the sample source is single. In future research, fragrant pear from multiple origins and growth conditions should be added as samples to further improve the generalization ability of the prediction model. In addition, the existing machine learning model will be continuously optimized, and new algorithms such as deep learning will be established to further enhance the application ability of dielectric spectroscopy to fruit quality detection, and relevant intelligent detection equipment will be developed to meet the needs of fruit enterprises and consumers for high-quality fruits.

## 5 Conclusion

In this study, a vector network analyser and coaxial probe were applied to detect dielectric parameters of fragrant pear in the frequency range of 0.1–26.5GHz. A new method for colour detection was proposed based on dielectric spectroscopy combined with machine learning models. The correlations between colour indexes (L^*^, a^*^ and b^*^) of fragrant pear and dielectric parameters (ϵ’ and ϵ’’) were poor at a single frequency, and it was difficult to predict the colour of fragrant pear under these conditions. Dielectric parameters at multiple frequencies should be used to predict the L^*^, a^*^, and b^*^ of fragrant pear. In comparison to the colour prediction model of fragrant pear established by full-frequency dielectric spectroscopy, the prediction accuracies of PLSR, SVR, and LSSVR models after UVE and SPA processing were improved. The optimal prediction model for L^*^ was SPA-PLSR (with R^2^ of 0.83, RMSE of 0.866, and RPD of 2.477), the optimal prediction model for a^*^ was UVE-PLSR (with R^2^ of 0.85, RMSE of 0.901, and RPD of 2.523), and the optimal prediction model for b^*^ was UVE-PLSR (with R^2^ of 0.73, RMSE of 0.895, and RPD of 1.973). In the future, more studies on quality detection methods of fragrant pear from multiple origins will be conducted, and novel algorithms will be explored to improve the practical application of dielectric spectroscopy.

## Data Availability

The original contributions presented in the study are included in the article/supplementary material. Further inquiries can be directed to the corresponding author.
